# Supplementary material for: Suppressed decomposition of organometal halide perovskites by impermeable electron-extraction layers in inverted solar cells
Source: Nat Commun. 2017 Jan 9;8:13938. doi: 10.1038/ncomms13938 (PMC5336555; doi:10.1038/ncomms13938)
Supplement: Supplementary Information — Supplementary Figures 1-8 and Supplementary References [file ncomms13938-s1.pdf]

# Supplementary Information

**Supplementary Fig. 1** studies the ALD growth of the $SnO_x$ layer on top of AZO, which has either been derived from an ALD process, and can therefore be considered as free of pinholes, or on top of an AZO layer prepared from a nanoparticle dispersion, where some porosity cannot be avoided. Details of the growth of AZO by ALD have been reported earlier.[1] The conformal nature of the ALD process is expected to yield some growth of $SnO_x$ inside the pores of the AZO layer. To analyze and quantify this effect, we performed Rutherford backscattering (RBS) measurements on the two samples shown in **Supplementary Fig. 1**. To this end, we have deposited nominally 20 nm (166 ALD cycles) of $SnO_x$ in one ALD process on top of both AZO layers. From the RBS spectra attributed to Sn, we are able to determine the number of Sn atoms per area, which is indicative of the amount of $SnO_x$ actually deposited. Interestingly, for the $SnO_x$ on top of the pin-hole-free ALD-AZO we measured $(44.5\pm0.2)\times10^{15}$ Sn-atoms per $cm^2$, which is in excellent agreement to earlier RBS measurements of layers with a thickness of 20 nm (determined by stylus profilometry). On the contrary, for the $SnO_x$ on top of the NP-AZO layer we found $(72.2\pm0.9)\times10^{15}$ Sn-atoms per $cm^2$, which is a factor of 1.62 higher than for the non-porous substrate. This result clearly supports the hypothesis of the ALD growth of the $SnO_x$ partially inside the pores of the nanoparticle derived AZO layer, which increases the total amount of $SnO_x$ deposited.

Moreover, the shoulder in the RBS spectrum of the Sn in case the $SnO_x$ layer has been grown on top of the AZO (NP) layer, is a direct result of a greater spatial variation of the Sn atoms in growth direction compared to the case where the $SnO_x$ layer has been grown on top of the AZO (ALD). This spatial variation originates from the infiltration of the $SnO_x$ into the porous AZO (NP) layer.

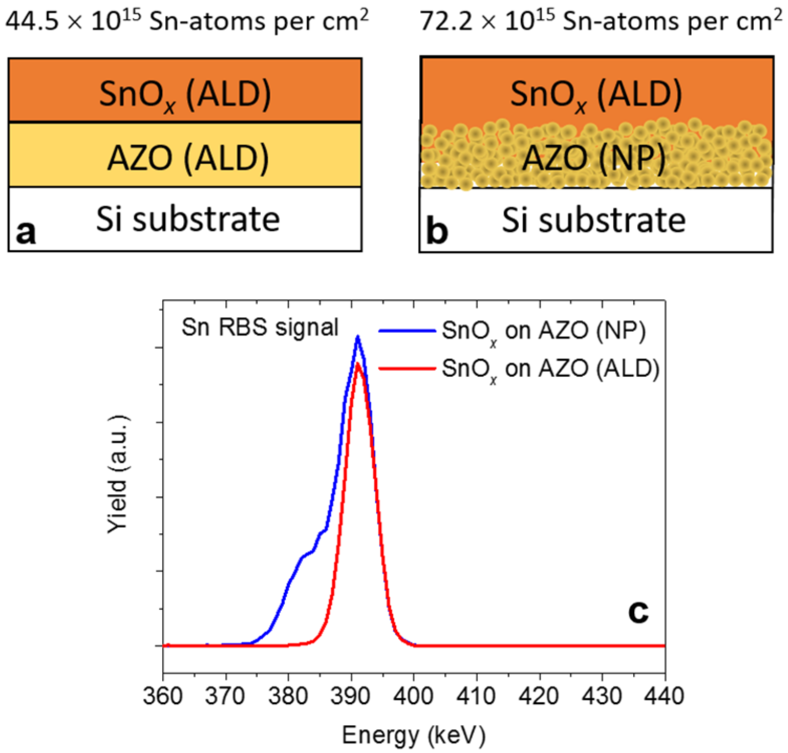


**Supplementary Figure 1: RBS study of the bi-layered AZO / $SnO_x$ EEL.** Layer sequence of samples used to study the ALD growth of the $SnO_x$ on top of an AZO layer grown by ALD, which is expected to have no pinholes (a), and on top of an AZO layer derived from a nanoparticle dispersion (b). The resulting RBS spectra attributed to Sn, which allow to determine the area density of Sn atoms (c).

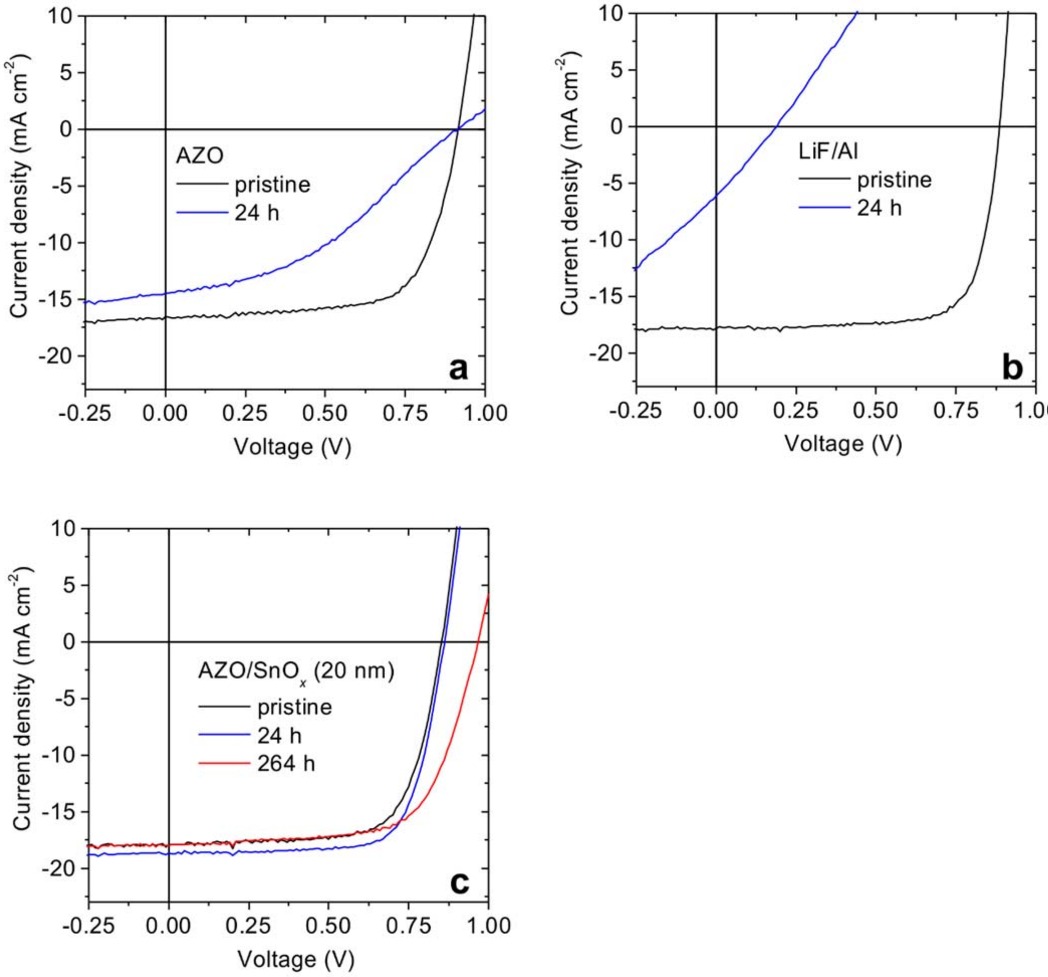


**Supplementary Figure 2: *J*/*V* characteristics of perovskite cells using different EELs in their pristine state and after some time in ambient air.** AZO (a), LiF/Al (b) and AZO/$SnO_x$ (c). Note, here the $SnO_x$ layer was 20 nm thick.

**Supplementary Fig. 2** shows the *J*/*V* characteristics of perovskite cells based on various electron extraction layers. The characteristics have been acquired in the pristine state and after storage in air for the time indicated. The extracted characteristics, i.e. $V_{oc}$, *FF*, $J_{sc}$, *PCE*, are discussed in the main manuscript (**Fig. 2**). In some batches of the perovskite, there is an increase of $V_{oc}$ on a time scale of several tens of hours. This is only observed in the AZO/$SnO_x$ samples, as the LiF/Al and the AZO cells already undergo strong degradation on the same time scale. The increase of the $V_{oc}$ on such a long time

scale may be associated with intrinsic changes of the defect structure in the perovskite, which is beyond the scope of the present work.

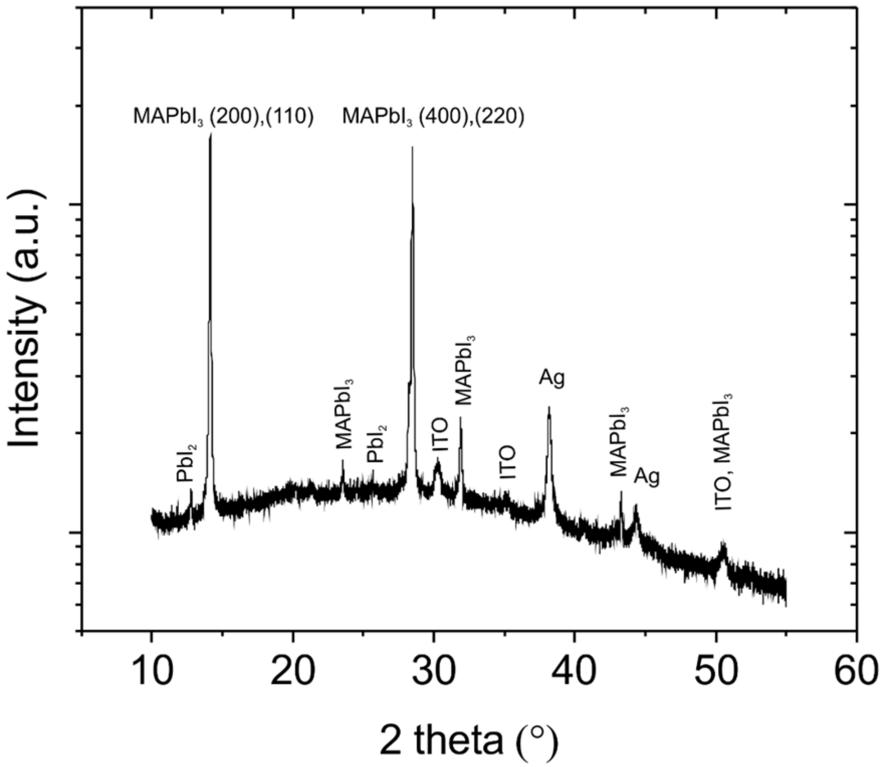


**Supplementary Figure 3: Logarithmic representation of the XRD spectrum of a fresh perovskite cell based on AZO as EEL with an assignment of the peaks to layers in the device.** The peak positions of $MAPbI_3$ (ref. 2), $PbI_2$ (ref. 3), ITO (ref. 4), and Ag (ref. 5), were taken from the respective references.

The survey XPS spectra for cells based on AZO, $SnO_x$ and bi-layered AZO/$SnO_x$ EELs, are shown in **Supplementary Fig. 4**. As expected, no signal due to the perovskite (e.g. Pb4d) can be detected, because the perovskite in these samples is covered by PCBM/AZO/Ag, or PCBM/$SnO_x$/Ag, or PCBM/AZO/$SnO_x$, respectively. Note, the position where the Pb4f signals would be located is overlaid by Zn3s and Sn4s signals as indicated in the figure, thus we refer to the Pb4d signal.

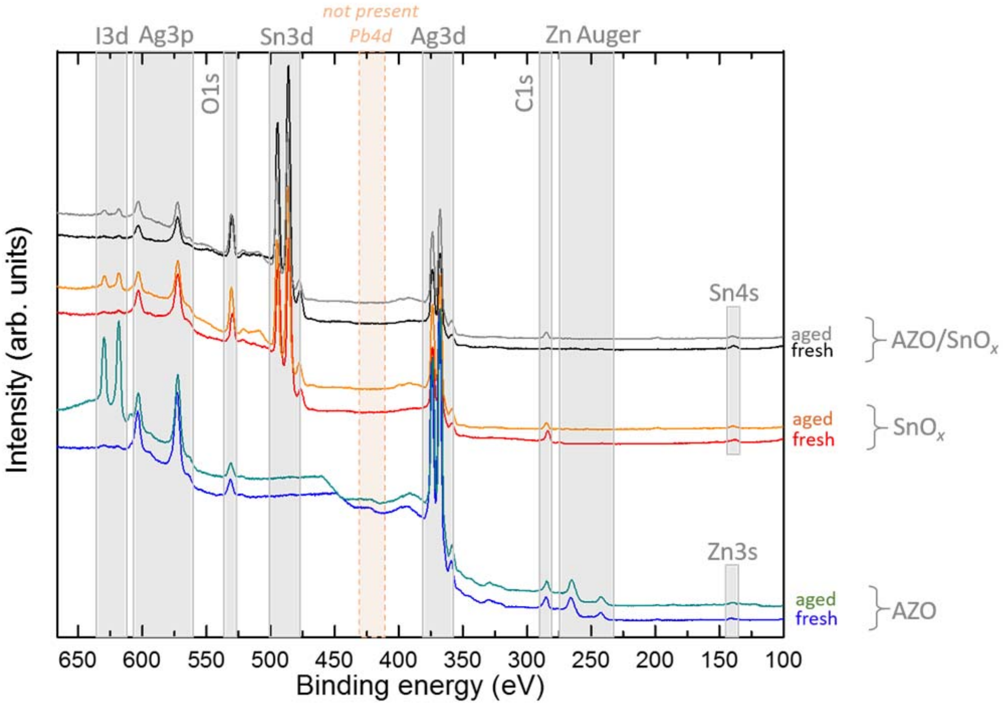


**Supplementary Figure 4: Survey photoemission spectra.** Spectra for cells based on AZO (fresh sample (blue), aged sample (green)), $SnO_x$ (fresh sample (red), aged sample (orange))and bi-layered AZO/$SnO_x$ (fresh sample (black), aged sample (grey)) EELs are shown, respectively. Samples were either fresh or aged for two days in air, respectively. The top Ag layer was only about 10 nm to see the metal-oxide underneath. The spectral region, where the signals due to electrons resulting from the respective atomic orbitals are found or expected are shaded in grey and labelled with the element and atomic orbital. The spectral region where the Pb4d signal would be expected is shaded on orange.

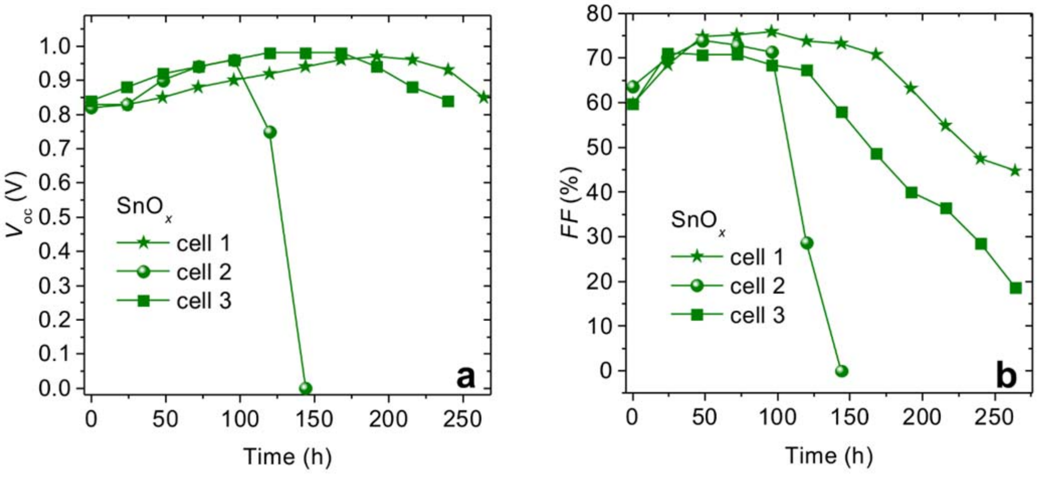


**Supplementary Figure 5: Stability of devices based on only SnO$_x$ as EEL.** (a) Characteristics of perovskite cells with 20 nm of ALD SnO$_x$ as EEL upon storage in ambient air (at 22°C, 60%rH). $V_{oc}$ vs. time (a) and *FF* vs. time (b).

The barrier properties of the AZO and AZO/SnO$_x$ EELs have been studied by using Ca-corrosion test. Detail of this test have been describe previously. [6] Specifically, we have used a setup as shown in **Supplementary Fig. 5a**. The substrate was a PET/ITO foil, which has an intrinsic water vapor transmission rate of $3\times10^{-2}$ gm$^{-2}$day$^{-1}$. On top of this substrate we have deposited the PCBM layer and the EEL in an identical way as in case of the solar cells. Subsequently, an array of Ca dots has been thermally evaporated (thickness 100 nm). The top-side of this stack has been encapsulated by a 200 nm thick $Al_2O_3$ gas diffusion barrier using ALD. The WVTR of the $Al_2O_3$ top-encapsulation is expected to be on the order of $10^{-6}$ gm$^{-2}$day$^{-1}$ (ref. 7). Thus only the water ingress from the bottom side through the substrate and the PCBM/EEL will be relevant for the corrosion of the Ca. While the Ca appears metallic in the pristine samples, the corrosion due to moisture will render the Ca transparent **Supplementary Fig. 5b**. Obviously, in the case of AZO the degradation of the Ca pads is substantially faster, as after 36h in air almost all the pads have vanished. Therefore the WVTR of the AZO layer does not provide any significant barrier against the ingress of moisture. On the contrary, in case of the AZO/SnO$_x$ EEL the Ca pads appear far less corroded. This corroborates the barrier functionality of the ALD grown SnO$_x$ layer on top of the AZO. It has to be noted that some statistical failure may be due to particle defects that may void the effectivity of the top encapsulation layer.[8]

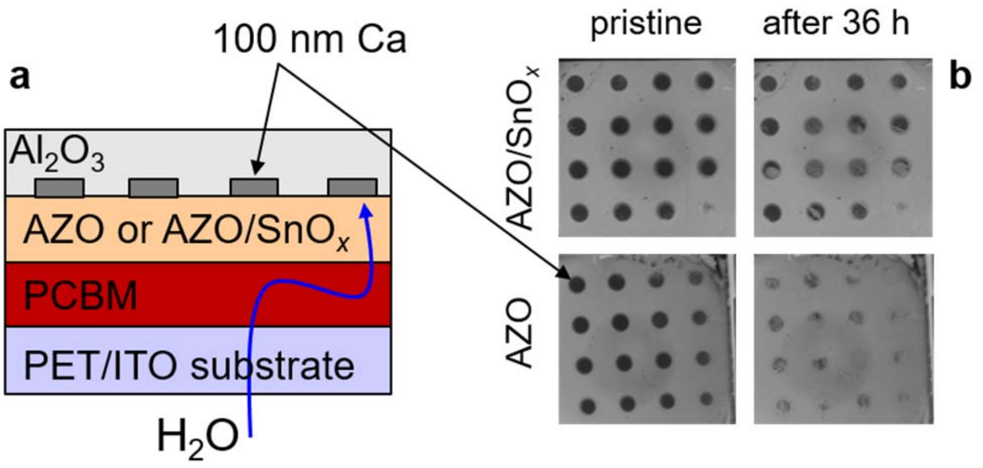


**Supplementary Figure 6: Permeation barrier functionality of AZO and AZO/SnO$_x$.** (a) Schematic of the Ca-test used to study the permeation barrier properties of the EEL. As substrate an ITO coated PET foil has been used (WVTR = $3\times10^{-2}$ gm$^{-2}$day$^{-1}$). Integrity of the Ca-dots in case of the AZO and AZO/SnO$_x$ samples in their pristine state and after exposure to ambient air for 36 h (b).

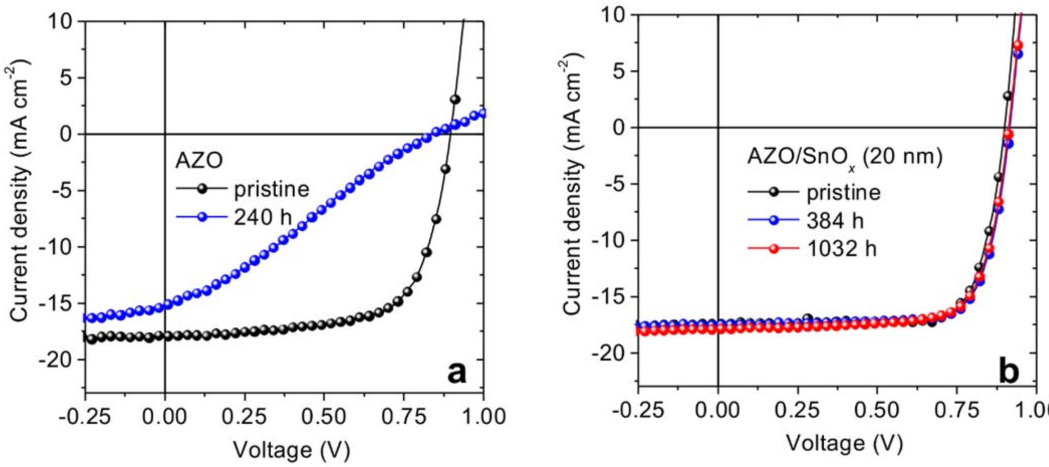


**Supplementary Figure 7: *J/V* characteristics of perovskite cells using different EELs in their pristine state and after aging at 60°C in inert $N_2$ atmosphere.** *J/V* characteristics of perovskite cells using AZO (a) and AZO/SnO$_x$ (b) as EEL.

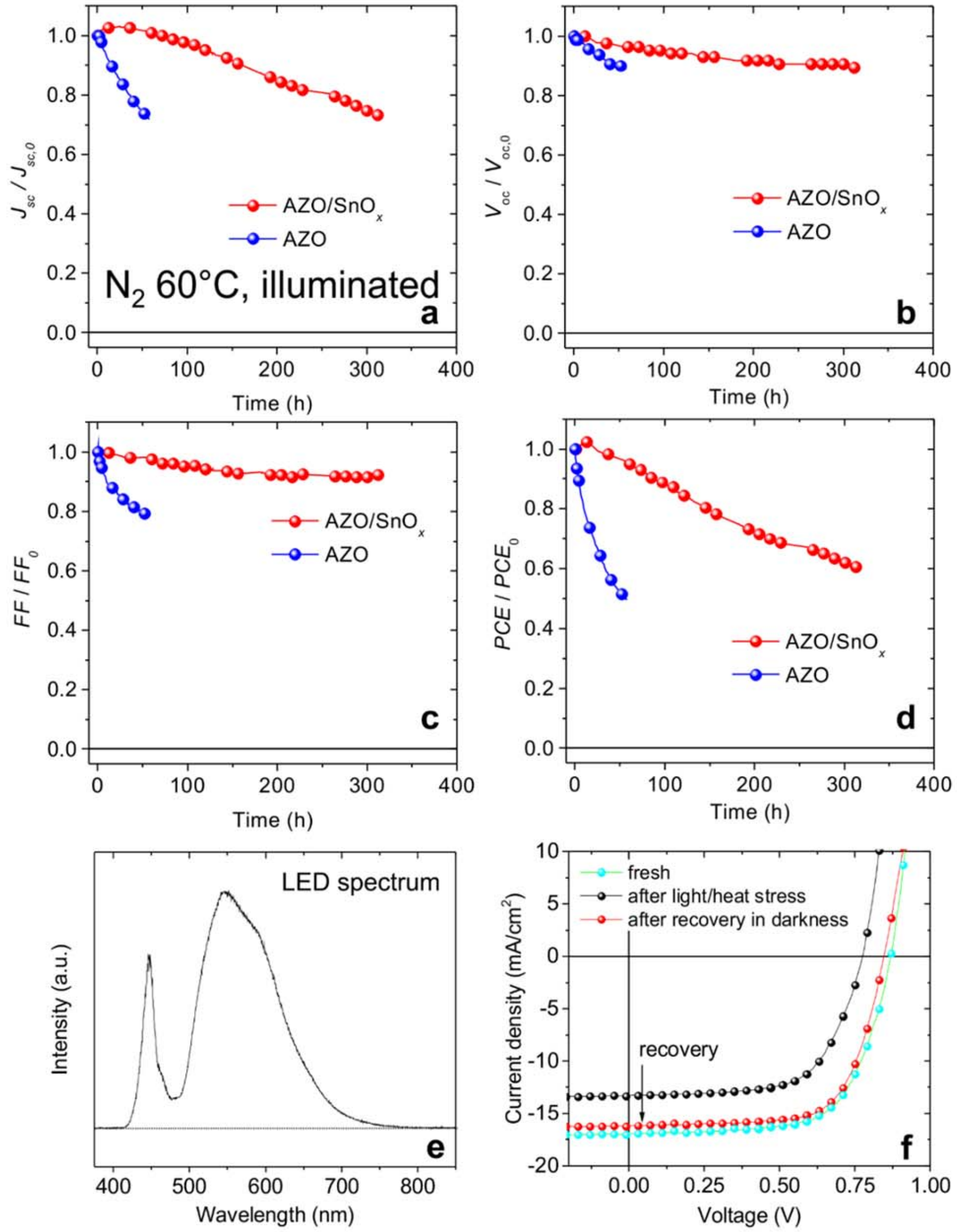


**Supplementary Figure 8: Characteristics of inverted perovskite cells with varied cathode electron extraction assemblies (AZO / $SnO_x$ / Ag, AZO / Ag) vs. time of storage at 60°C in nitrogen atmosphere and simultaneous illumination**. A white LED has been used for illumination to achieve the same $J_{sc}$ as under the solar simulator. $J_{sc}$ (a), $V_{oc}$ (b), *FF* (c), and *PCE* (d). $J_{sc,0}$, $V_{oc,0}$, $FF_0$, and $PCE_0$ are the characteristics of the cells before stressing. In this set of samples, the thickness of the ALD $SnO_x$ layer was 20 nm and that of the Ag electrode was 100 nm. The characteristics have been determined from the *J/V* measurements in reverse direction. Spectrum of the white LED used for the light-soaking stress test under concomitant heating (e). *J/V* characteristics

of an AZO/$SnO_x$/Ag cell, non-stressed (fresh), stressed for 300 hours (light/heat), and subsequently recovered in in darkness at room temperature for nine days (in the glove box).

## Supplementary References


1. Behrendt A*, et al.* Highly Robust Transparent and Conductive Gas Diffusion Barriers Based on Tin Oxide. *Adv Mater* **27**, 5961-5967 (2015).

2. Safdari M, Fischer A, Xu B, Kloo L, Gardner JM. Structure and function relationships in alkylammonium lead(II) iodide solar cells. *Journal of Materials Chemistry A* **3**, 9201-9207 (2015).

3. Zhou Y*, et al.* Growth control of compact $CH_3NH_3PbI_3$ thin films via enhanced solid-state precursor reaction for efficient planar perovskite solar cells. *Journal of Materials Chemistry A* **3**, 9249-9256 (2015).

4. Sunde TOL*, et al.* Transparent and conducting ITO thin films by spin coating of an aqueous precursor solution. *J Mater Chem* **22**, 15740-15749 (2012).

5. Kato Y, Ono LK, Lee MV, Wang S, Raga SR, Qi Y. Silver Iodide Formation in Methyl Ammonium Lead Iodide Perovskite Solar Cells with Silver Top Electrodes. *Advanced Materials Interfaces* **2**, 1500195 (2015).

6. Hoffmann L, Theirich D, Hasselmann T, Räupke A, Schlamm D, Riedl T. Gas permeation barriers deposited by atmospheric pressure plasma enhanced atomic layer deposition. *J Vac Sci Technol A* **34**, 01A114 (2016).

7. Meyer J*, et al.* $Al_2O_3$/$ZrO_2$ Nanolaminates as Ultrahigh Gas-Diffusion Barriers-A Strategy for Reliable Encapsulation of Organic Electronics. *Adv Mater* **21**, 1845-1849 (2009).

8. Behrendt A, Meyer J, van de Weijer P, Gahlmann T, Heiderhoff R, Riedl T. Stress Management in Thin-Film Gas-Permeation Barriers. *Acs Appl Mater Inter* **8**, 4056-4061 (2016).
